# Diagnostic Concordance Characteristics of Oral Cavity Lesions

**DOI:** 10.1155/2013/785929

**Published:** 2013-12-25

**Authors:** Ufuk Tatli, Özgür Erdoğan, Aysun Uğuz, Yakup Üstün, Yaşar Sertdemır, İbrahim Damlar

**Affiliations:** ^1^Department of Oral and Maxillofacial Surgery, Faculty of Dentistry, Çukurova University, Saricam-Balcali, 01330 Adana, Turkey; ^2^Department of Pathology, Faculty of Medicine, Çukurova University, 01330 Adana, Turkey; ^3^Private Practice in Oral and Maxillofacial Surgery, 01120 Adana, Turkey; ^4^Department of Biostatistics, Faculty of Medicine, Çukurova University, 01330 Adana, Turkey; ^5^Department of Oral and Maxillofacial Surgery, Faculty of Dentistry, Mustafa Kemal University, 31100 Hatay, Turkey

## Abstract

*Purpose*. The objective of this study was to evaluate the diagnostic concordance characteristics of oral cavity lesions by comparing the clinical diagnosis of the lesions with the histopathologic diagnosis. *Material and Method*. A retrospective analysis was conducted on the patients, who were admitted with oral cavity pathology and underwent biopsy procedure between 2007 and 2011. The oral cavity lesions were classified into 6 different groups as odontogenic cysts, nonodontogenic cysts, odontogenic tumors, nonodontogenic tumors, malignant tumors, and precancerous lesions in accordance with the 2005 WHO classification. The diagnoses were also recategorized into 3 groups expressing prognostic implications as benign, precancerous, and malignant. The initial clinical diagnoses were compared with the histopathologic diagnoses. Data were analyzed statistically. *Results*. A total of 2718 cases were included. Histopathologic diagnosis did not match the clinical diagnosis in 6.7% of the cases. Nonodontogenic tumors and malignant tumors had the highest misdiagnosis rates (11.5% and 9%, resp.), followed by odontogenic tumors (7.7%), precancerous lesions (6.9%), and odontogenic cysts (4.4%). Clinicians were excelled in diagnosis of benign and precancerous lesions in clinical setting. *Conclusion*. The detailed discordance characteristics for each specific lesion should be considered during oral pathology practice to provide early detection without delay.

## 1. Introduction

Oral cavity is a complex district of the head and neck region consisting of various structures such as teeth, jaws, tongue, salivary glands, and soft and hard palate. The cavity hosts a wide variety of cysts and neoplasms of both odontogenic and nonodontogenic origins and sometimes it can be very difficult to diagnose these lesions clinically [1]. The diagnosis and treatment of oral cavity lesions are integral parts of oral health care. Histopathologic examination is regarded as the gold standard in diagnostic oral pathology to confirm the clinical diagnosis [2]. It is known that early detection and treatment have a significant role in the improvement of the survival rate and life quality of patients [3]. Hence, the initial clinical diagnosis made by clinicians must be accurate and should not have missed any precancerous or malignant features. Thus, the assessment of the concordance between clinical and histopathologic diagnosis of oral cavity lesions is crucial. In the literature, little data is available about the concordance of oral cavity lesions [2].

The aim of this study was to evaluate the diagnostic concordance characteristics of oral cavity lesions in detail by comparing the clinical diagnosis of the lesions with the histopathologic diagnosis. The demographic characteristics of the lesions were also compared with those from other parts of the world.

## 2. Material and Methods

A retrospective chart review was conducted on patients, who underwent biopsy procedure at the Department of Oral and Maxillofacial Surgery, Faculty of Dentistry, Çukurova University (Adana, Turkey), between 2007 and 2011. Due to the retrospective nature of this study, it was granted an exemption in writing by the ethical review committee of Çukurova University Medical Scientific Research Center. We followed the guidelines of Helsinki Declaration in the present study. Data including age, gender, anatomic localization of the lesion, and clinical and histopathologic diagnosis were recorded. Patients, with lack of any of these data mentioned above, were excluded. The recurring or reoperated lesions were considered as one lesion. Each initial clinical diagnosis was made by a consultant oral and maxillofacial surgeon, who attended the biopsy procedure.

The oral cavity lesions were classified into 6 different groups as odontogenic cysts (OCs), nonodontogenic cysts (NOCs), odontogenic tumors (OTs), nonodontogenic tumors (NOTs), malignant tumors (MTs), and precancerous lesions (PLs). Furthermore, the clinical and histopathological diagnoses were also recategorized into 3 groups expressing prognostic implications as benign, precancerous, and malignant. The latest WHO histological classification of tumors (2005) was used to subcategorize oral cavity lesions. One of the main modifications found in the newest edition was the addition of the odontogenic keratocyst as a benign but locally aggressive epithelial odontogenic tumor, which has been renamed as keratocystic odontogenic tumor (KCOT).

The clinical diagnoses of the lesions were compared with the histopathologic diagnoses of the specimens. The frequency and characteristics of clinical misdiagnosis were determined for each specific lesion. Data were analyzed using SPSS 18.0 software (SPSS Inc., Chicago, IL). Cohen's kappa statistic was used to determine the concordance between clinical and histopathologic diagnosis and *P* values smaller than 0.05 were considered to be statistically significant. Cohen's kappa coefficient (*κ*) is a statistical measure of interrater agreement for categorical items. It was used to measure the agreement between surgeon and pathologist.

## 3. Results

**Figure 1:**
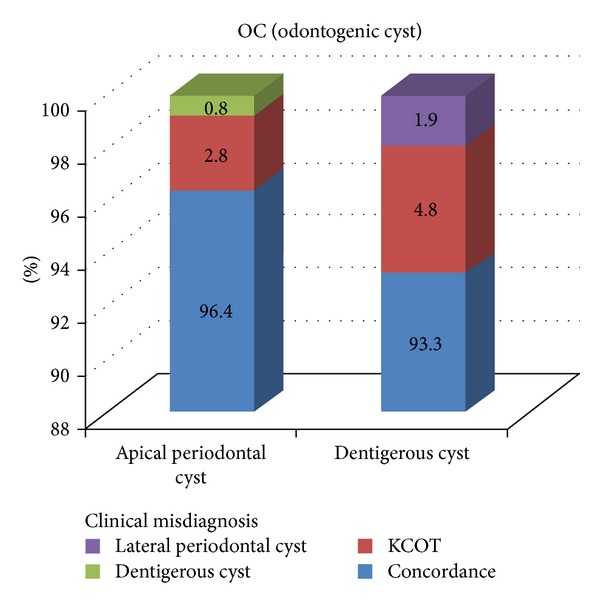
The detailed characteristics of diagnostic discordance of odontogenic cysts. KCOT: keratocystic odontogenic tumor.

**Figure 2:**
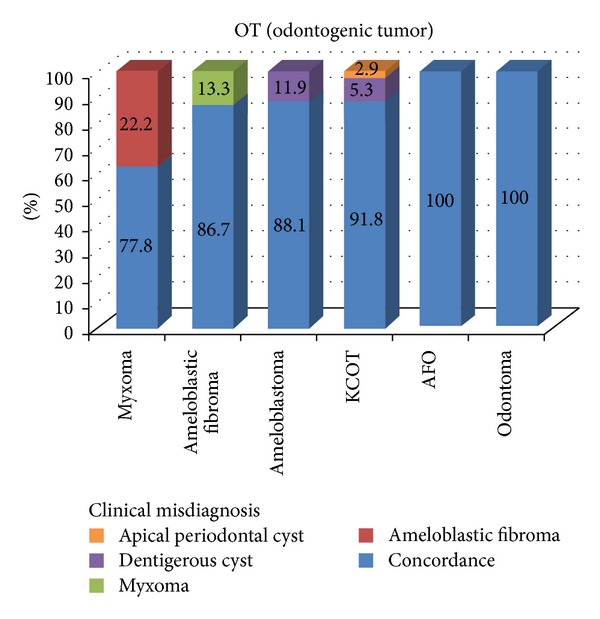
The detailed characteristics of diagnostic discordance of odontogenic tumors. KCOT: keratocystic odontogenic tumor, AFO: ameloblastic fibroodontoma.

**Figure 3:**
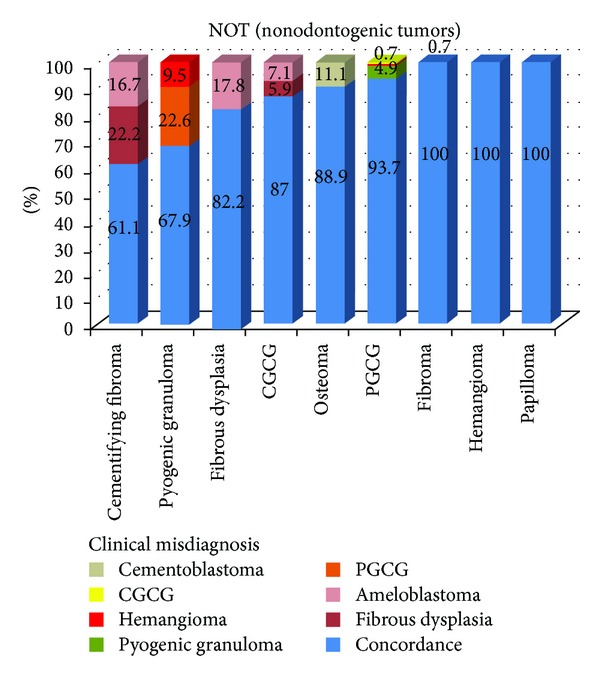
The detailed characteristics of diagnostic discordance of nonodontogenic tumors. CGCG: central giant cell granuloma, PGCG: peripheral giant cell granuloma.

**Figure 4:**
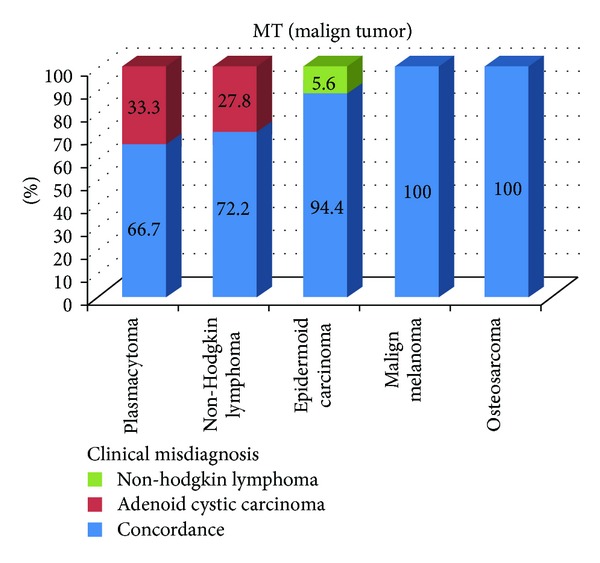
The detailed characteristics of diagnostic discordance of malignant tumors.

**Figure 5:**
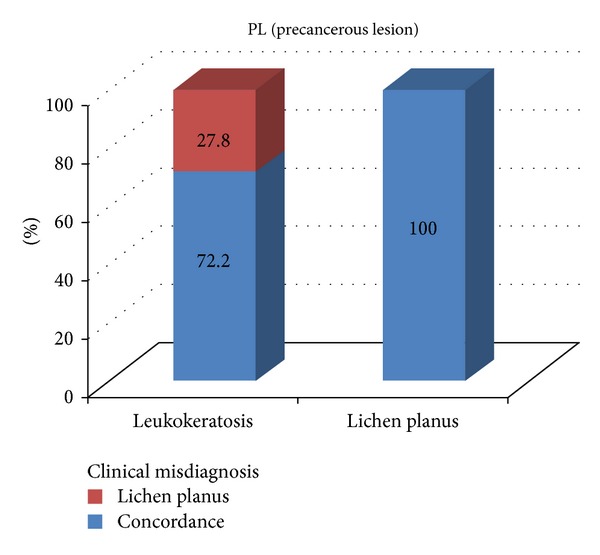
The detailed characteristics of diagnostic discordance of precancerous lesions.

Out of 4170 patients, 2718 cases (1372 males, 1346 females) fulfilled the inclusion criteria and were included in the study. The mean age of the patients was 38.3 ± 16.2 years (range, 5–82 years). The distributions of the lesions according to anatomic localization, gender, and age was shown in Tables 1 and 2. The statistical measure of concordance between clinical and histopathologic diagnosis of oral cavity lesions were shown in Table 3. The concordance characteristics of oral cavity lesions in terms of localization and gender were shown in Tables 4 and 5. The detailed misdiagnosis rates for each lesion were illustrated in Figures 1–5. In 6.7% of the cases, the histopathologic diagnosis did not confirm surgeon's initial clinical diagnosis. In terms of prognostic implication, 99.8% rate of agreement between clinicians and pathologists was evident for lesions considered clinically benign; however, the consensus was 100% for clinical diagnosis of precancerous and malign lesions (Table 6).

### 3.1. Patients with OCs

The occurrence rate of OCs in males was 1.36 times greater than in females (Table 1). In terms of anatomic localization, there was increased propensity for OCs to occur in the mandible (about 1.19 times greater than in the maxilla) (Table 2). The overall frequency of diagnostic concordance for the OCs was 95.6% (*κ* = 0.891) (Table 3). The detailed characteristics of diagnostic discordance were illustrated in Figure 1. Among apical periodontal cysts, 2.8% of cases were clinically misdiagnosed as KCOT, and 0.8% of cases were as dentigerous cyst. Among dentigerous cysts, 4.8% of cases were clinically misdiagnosed as KCOT, and 1.9% of cases were as lateral periodontal cyst.

### 3.2. Patients with NOCs

The occurrence rate of NOCs in males was 1.19 times greater than in females (Table 1). There was increased propensity for NOCs to occur in the maxilla (about 1.46 times greater than in mandible) (Table 2). Unlike other lesions of the oral cavity, the histopathologic diagnosis confirmed the initial diagnosis in all NOC cases.

### 3.3. Patients with OTs

The occurrence rate of OTs in females was 1.14 times greater than in males (Table 1). There was increased propensity for OTs to occur in the mandible (about 1.7 times more than in the maxilla) (Table 2). Among the OT cases, the frequency of discordance between the clinical and histopathologic diagnosis was 7.7% (*κ* = 0.889) (Table 3). The detailed characteristics of diagnostic discordance for OTs were illustrated in Figure 2. Among OTs, ameloblastic fibroodontoma (AFO) and odontoma cases had no diagnostic discordance.

### 3.4. Patients with NOTs

The occurrence rate of NOTs in females was 1.42 times greater than in males (Table 1). A total of 608 cases were observed in the jawbones (including 284 cases in maxilla and 324 cases in mandible) whereas only 46 cases were observed in the other locations (including cheek mucosa, tongue, lips, salivary glands, and floor of mouth). There was increased propensity for NOTs to occur in jaw bones (about 13.21 times greater than in other localizations) (Table 2). The histopathologic diagnosis did not confirm the initial diagnosis in 11.5% of NOTs (*κ* = 0.849), which corresponds to the highest discordance rate in overall of oral cavity lesions. The detailed characteristics of diagnostic discordance for NOTs were illustrated in Figure 3. Among NOTs, cementifying fibroma (CF) and pyogenic granuloma (PG) had the highest discordance rates (38.9% and 32.1%, resp.). On the other hand, fibroma, hemangioma, and papilloma had no diagnostic discordance (Table 3).

### 3.5. Patients with MTs

The occurrence rate of MTs in females was 2.06 times greater than in males (Table 1). There was increased propensity for MTs to occur in the mandible (about 1.52 times greater than in the maxilla) (Table 2). The histopathologic diagnosis did not confirm the initial diagnosis in 9% of MTs (*κ* = 0.798) (Table 3). The detailed characteristics of diagnostic discordance for MTs were illustrated in Figure 4.

### 3.6. Patients with PLs

There was increased propensity for PLs to occur in females (1.6 times greater than in males) (Table 1). All of the PL cases were located in regions other than the jawbones. In 6.9% of PLs, the initial diagnoses did not match the final diagnosis (*κ* = 0.796) (Table 3). The detailed characteristics of diagnostic discordance for MTs were illustrated in Figure 5.

## 4. Discussion

Regular epidemiologic monitoring of the oral cavity lesions within a population is important for preventive approaches and future planning. According to our demographic data, OCs and NOCs had higher tendency to occur in males. On the contrary, OTs, NOTs, MTs, and PLs were more commonly seen in females. Overall, the male to female ratio for oral cavity lesions was found to be 1.02 : 1. Our general finding was that MTs of oral cavity were observed in the sixth decade of life, while the other oral cavity lesions were observed in the fourth and fifth decade of life in the Turkish population.

Valid demographic comparison between studies among different parts of the world is difficult because most of the studies were conducted in accordance with the 1992 WHO classification, and only limited numbers of studies have been available in accordance with the 2005 WHO classification [4–7]. The present study is the first report concerning the demographic characteristics of the oral cavity lesions among the Turkish population based on the 2005 WHO classification. The inclusion of KCOT occupies a preponderant place in the prevalence of OTs in epidemiological studies, because it is a relatively common tumor of the jaws. In the present study, odontogenic tumors accounted for 11.9% of the included pathologies, which is higher than that in many studies [5, 8, 9] and lower than some studies from Africa [10, 11]. We found KCOT to be the most common OT consistent with corresponding data reported by authors from Brazil [4] and China [5] that followed 2005 WHO classification of tumors. On the other hand, Varkhede et al. [6] from India reported ameloblastoma to be the most frequent OT followed by KCOT and odontoma.

In our study, OTs were more frequently observed in females with a female to male ratio of 1.15. In terms of female to male ratio, similar results were reported from UK [8], Brazil [9], Mexico [12], and Chile [13]. However studies from Nigeria [10], Libya [11], and China [5] reported male predominance in OTs with a male to female ratio of 1.01, 1.31, and 1.35, respectively. In our study, the ratio of mandible to maxilla regarding the incidence of OTs was 1.7 which was lower than the reported series from Brazil [9], Libya [11], China, [5] and Nigeria [10] (the ratio of mandibula to maxilla was 2, 2.1, 3.5, and 4.1, resp.). In the present study, 3.5% of KCOT cases were associated with Gorlin-Goltz syndrome. The studies from Japan [7], Iran [14], and Chile [13] reported higher frequency than our results (6%, 8.1%, and 15.4%, resp.).

Malignancies involving oral mucosa and pharynx rank the sixth overall in the world [15]. Our study indicated the rate of malignant cases as 5.3%. This result is similar to the studies from Singapore [16] and UK [8], which reported the rate of malignant cases as 5.2% and 5.4%, respectively. In the present study, epidermoid carcinoma was the most frequently diagnosed malignant lesion of the oral cavity in accordance with the previous studies [15–17].

Most of the clinicians act on an initial clinically diagnosis before embarking on a biopsy to establish a tissue diagnosis [18]. This can be beneficial for beginning treatment without delay if the initial clinical diagnosis is accurate. There is a data deficiency on the assessment of the diagnostic concordance between the clinical and histopathologic diagnosis of oral cavity lesions [2]. Thus, in the present study, we sought to determine the frequency of discordance between the clinical and histopathologic diagnosis. According to our results, clinicians had slightly less discordance rates for the lesions which were in other localizations (4.3%) compared with maxilla (6.7%) and mandible (7%). This might be due to the specific characteristics of locations such as tongue, cheek mucosa, and salivary glands. According to the gender characteristics of concordance of the oral cavity lesions, clinicians had slightly higher discordance rates for the lesions of the female patients (7.7%) compared with male patients (5.7%). This might be due to the increased incidence of the oral cavity lesions in females except for OCs and NOCs, which had the fewest discordance rates.

Odontogenic cysts had significantly fewer misdiagnosis rates (4.4%) compared with other oral pathologies. This might have been due to the fact that that OCs were commonly found lesions and had a limited range of subgroups. So clinicians were thought to be familiar with OCs. In terms of OTs, it was noted that the rate of misdiagnosis of ameloblastoma as dentigerous cyst was 11.9%. Clinical characteristics of unicystic ameloblastoma can show similarities with odontogenic cysts [19]. In the present study, the concordance rate of KCOT cases was 91.8%. This result was higher than that defined by Güler et al. [20] who reported a diagnostic concordance of 39.5% for KCOTs in their clinical study.

A significant finding of our study was that there was no diagnostic discordance with regard to NOCs of the oral cavity. This might be due to the developmental characteristics of these lesions in specific locations such as salivary glands, palatal bone, or soft tissues, hence minimizing the risk of misdiagnosis of these lesions.

Nonodontogenic tumors had the highest inaccurate initial diagnosis rate with a ratio of 11.5%. This might have been due to the fact that most of NOTs in oral cavity had similar clinical and radiographic features. Thus, it was thought that among oral cavity lesions NOTs had challenging characteristics to diagnose for clinicians.

Among MLs, plasmacytomas and Non-Hodgkin lymphomas had high misdiagnosis rates (33.3% and 27.8%, resp.). This might be due to the fact that these lesions are among the relatively rare pathologic lesions in oral cavity. Thus, these diagnoses did not become the clinicians' first diagnostic choice. It is known that plasmacytoma has some systemic symptoms such as renal failure, hypercalcemia, anemia, and thrombocytopenia [21]. The efficiencies of fine-needle aspiration cytology and brush cytology were defined in the literature [22, 23]. Fontes et al. [24] reported that cytopathology was a reliable method with 83.1% sensitivity and 100% specificity for patients who require the diagnosis of suspected squamous cell carcinoma for starting treatment. Thus, when dealing with such pathologic lesions with high misdiagnosis rates, adjunct techniques such as fine-needle aspiration cytology, brush cytology, or blood-urine sample analysis should be considered when appropriate.

Among PLs, 27.8% of leuckkeratoses were misdiagnosed as lichen planus. It is known that white soft tissue lesions are difficult to diagnose and both lesions with negative Nikolsky's sign are expected candidates for misdiagnosis.

The present study showed that clinicians were excelled in the diagnosis of benign and precancerous lesions in clinical setting. On the other hand, clinicians missed 5 instances (3.47% of all malign lesions) of malignancy clinically (Table 6). Diagnostic concordance between general dental practitioners and specialists was reported without detailed discordance rates in the literature [2, 25]. But, to our knowledge, this is the first study concerning the concordance characteristics between clinical and histopathologic diagnosis of oral cavity lesions with detailed rates and statistical comparison.

In conclusion, the reported diagnostic failure rates may be regarded as differential diagnosis percentages of oral cavity lesions. The detailed discordance characteristics for each specific lesion should be considered during oral pathology practice to provide early detection without delay. Further studies with higher number of samples are necessary in order to make more clear comments about clinical misdiagnosis characteristics of oral cavity lesions.

## Figures and Tables

**Table 1:** Distribution of oral cavity lesions according to gender and age (mean ± SD).

Group	Male		Female		Total	
	*N* (%)	Age	*N* (%)	Age	*N *	Age
OC	828 (57.7)	36.5 ± 17.8	606 (42.3)	36.1 ± 17.9	1434	36.3 ± 17.8
NOC	49 (54.4)	42.1 ± 10.5	41 (45.6)	44.1 ± 7.1	90	43.1 ± 9.1
OT	151 (46.6)	35.1 ± 12.1	173 (53.4)	35.1 ± 14.2	324	35.1 ± 13.2
NOT	270 (41.3)	37.9 ± 14.3	384 (58.7)	40.8 ± 13.3	654	39.6 ± 13.7
MT	47 (32.6)	52.5 ± 9.2	97 (67.4)	53.5 ± 9.8	144	53.2 ± 9.5
PL	27 (37.5)	45.4 ± 8.2	45 (62.5)	47.6 ± 9.8	72	46.8 ± 9.2

Total	1372 (50.5)	37.5 ± 16.3	1346 (49.5)	39.2 ± 16.1	2718	38.4 ± 16.2

OC: odontogenic cyst, NOC: nonodontogenic cyst, OT: odontogenic tumor, NOT: nonodontogenic tumor, MT: malign tumor, and PL: precancerous lesion.

**Table 2:** Distribution of oral cavity lesions according to anatomic localization and age (mean ± SD).

Group	Maxilla		Mandible		Other	
	*N* (%)	Age	*N* (%)	Age	*N* (%)	Age
OC	652 (45.5)	36.1 ± 18.5	782 (54.5)	36.5 ± 17.3	—	—
NOC	44 (48.9)	43.1 ± 6.7	30 (33.3)	45.6 ± 10.2	16 (17.8)	37.9 ± 11.2
OT	120 (37.1)	30.7 ± 13.1	204 (62.9)	37.6 ± 12.6	—	—
NOT	284 (43.4)	38.2 ± 12.9	324 (49.5)	40.4 ± 14.4	46 (7.1)	42.6 ± 13.4
MT	54 (37.5)	53.9 ± 9.6	82 (56.9)	52.1 ± 9.1	8 (5.6)	59.7 ± 11.8
PL	—	—	—	—	72 (100)	46.8 ± 9.2

Total	1154 (42.5)	37.2 ± 16.6	1422 (52.3)	38.6 ± 16.1	142 (5.2)	45.2 ± 11.9

Abbreviations were the same as in Table 1. The localization termed “other” contains cheek mucosa, tongue, lips, salivary glands, and floor of mouth.

**Table 3:** Statistical measure of concordance between clinical and histopathologic diagnosis in oral cavity lesions.

Histopathologic diagnosis	Concordance	Discordance	Total	*κ*	*P*
	*N* (%)	*N* (%)	*N*		
Odontogenic cysts	1370 (95.6)	64 (4.4)	1434	.891	.000
Nonodontogenic cysts	90 (100)	0	90	1	.000
Odontogenic tumors	299 (92.3)	25 (7.7)	324	.889	.000
Nonodontogenic tumors	579 (88.5)	75 (11.5)	654	.849	.000
Malign tumors	131 (91)	13 (9)	144	.798	.000
Precancerous lesions	67 (93.1)	5 (6.9)	72	.796	.000

Total	2536 (93.3)	182 (6.7)	2718	.918	.000

**Table 4:** Localization characteristics of concordance between clinical and histopathological diagnosis in oral cavity lesions.

Localization	Concordance	Discordance	Total	*κ*	*P*
	*N* (%)	*N* (%)	*N *		
Maxilla	1077 (93.3)	77 (6.7)	1154	0.915	.000
Mandibula	1323 (93)	99 (7)	1422	0.913	.000
Other	136 (95.7)	6 (4.3)	142	0.945	.000

Total	2536 (93.3)	182 (6.7)	2718	0.918	.000

**Table 5:** Gender characteristics of concordance between clinical and histopathological diagnosis in oral cavity lesions.

Gender	Concordance	Discordance	Total	*κ*	*P*
	*N* (%)	*N* (%)	*N *		
Male	1294 (94.3)	78 (5.7)	1372	0.926	.000
Female	1242 (92.3)	104 (7.7)	1346	0.909	.000

Total	2536 (93.3)	182 (6.7)	2718	0.918	.000

**Table 6:** The concordance rate for the implied prognosis of clinical diagnosis against histopathologic diagnosis in oral cavity lesions.

Clinical diagnosis	Histopathologic diagnosis			
	Benign	Premalign	Malign	Total
Benign	2502 (99.8%)	0	5 (0.2%)	2507
Premalign	0	72 (100%)	0	72
Malign	0	0	139 (100%)	139

Total	2502 (92.1%)	72 (2.6%)	144 (5.3%)	2718
